# Regulation of neural stem cell proliferation and survival by protein arginine methyltransferase 1

**DOI:** 10.3389/fnins.2022.948517

**Published:** 2022-11-10

**Authors:** Misuzu Hashimoto, Kaho Takeichi, Kazuya Murata, Aoi Kozakai, Atsushi Yagi, Kohei Ishikawa, Chiharu Suzuki-Nakagawa, Yoshitoshi Kasuya, Akiyoshi Fukamizu, Tsutomu Nakagawa

**Affiliations:** ^1^Laboratory of Biological Chemistry, Faculty of Applied Biological Sciences, Gifu University, Gifu, Japan; ^2^Laboratory Animal Resource Center in Transborder Medical Research Center, Faculty of Medicine, University of Tsukuba, Tsukuba, Japan; ^3^Department of Biochemistry and Molecular Pharmacology, Graduate School of Medicine, Chiba University, Chiba, Japan; ^4^Life Science Center for Survival Dynamics, Tsukuba Advanced Research Alliance, University of Tsukuba, Tsukuba, Japan; ^5^World Premier International Research Center Initiative, International Institute for Integrative Sleep Medicine, University of Tsukuba, Tsukuba, Japan; ^6^AMED-CREST, Japan Agency for Medical Research and Development, Tokyo, Japan

**Keywords:** PRMT1, neural stem cell, apoptosis, development, p53

## Abstract

Protein arginine methyltransferase 1 (PRMT1), a major type I arginine methyltransferase in mammals, methylates histone and non-histone proteins to regulate various cellular functions, such as transcription, DNA damage response, and signal transduction. PRMT1 is highly expressed in neural stem cells (NSCs) and embryonic brains, suggesting that PRMT1 is essential for early brain development. Although our previous reports have shown that PRMT1 positively regulates oligodendrocyte development, it has not been studied whether PRMT1 regulates NSC proliferation and its survival during development. To examine the role of PRMT1 in NSC activity, we cultured NSCs prepared from embryonic mouse forebrains deficient in PRMT1 specific for NSCs and performed neurosphere assays. We found that the primary neurospheres of PRMT1-deficient NSCs were small and the number of spheres was decreased, compared to those of control NSCs. Primary neurospheres deficient in PRMT1 expressed an increased level of cleaved caspase-3, suggesting that PRMT1 deficiency-induced apoptosis. Furthermore, p53 protein was significantly accumulated in PRMT1-deficient NSCs. In parallel, p53-responsive pro-apoptotic genes including *Pmaip1* and *Perp* were upregulated in PRMT1-deficient NSCs. p53-target p21 mRNA and its protein levels were shown to be upregulated in PRMT1-deficient NSCs. Moreover, the 5-bromo-2′-deoxyuridine (BrdU) incorporation assay showed that the loss of PRMT1 led to cell cycle defects in the embryonic NSCs. In contrast to the above *in vitro* observations, NSCs normally proliferated and survived in the fetal brains of NSC-specific PRMT1-deficient mice. We also found that *Lama1*, which encodes the laminin subunit α1, was significantly upregulated in the embryonic brains of PRMT1-deficient mice. These data implicate that extracellular factors provided by neighboring cells in the microenvironment gave a trophic support to NSCs in the PRMT1-deficient brain and recovered NSC activity to maintain brain homeostasis. Our study implies that PRMT1 plays a cell-autonomous role in the survival and proliferation of embryonic NSCs.

## Introduction

Mammalian brain tissues are originally derived from neural stem cells (NSCs) proliferation and differentiation (Ohtsuka and Kageyama, 2019). During brain development, NSCs self-renew and expand to populate the central nervous system (CNS) (Ohtsuka and Kageyama, 2019). After proliferation, they differentiate into neurons, oligodendrocytes, and astrocytes to make a complex cell network and perform high-order functions in the brain (Ohtsuka and Kageyama, 2019). A small number of NSCs are also distributed in adult brains and are suggested to be important for neuronal regeneration (Bond et al., 2015). However, it is still unclear how NSCs proliferate before differentiating into functional cells. Recent studies attempt to use NSCs for stem cell therapy for various complications such as spinal cord injury (Yousefifard et al., 2016; Marsh and Blurton-Jones, 2017; Kitagawa et al., 2022). Therefore, understanding the molecular control on how NSC proliferation and viability are regulated is an important topic.

Protein arginine methylation is one of the major post-translational modifications mediated by a PRMT family (Bedford and Clarke, 2009; Blanc and Richard, 2017). The substrates of protein arginine methyltransferases (PRMTs) vary from histone and non-histone proteins, and their methylation controls cell survival, transcriptional regulation, and signal transduction (Bedford and Clarke, 2009). Type I PRMTs including PRMT1, 2, 3, 6, 8, CARM1, and METTL23 are responsible for the monomethylation and asymmetric dimethylation of arginine. On the other hand, type II PRMTs such as PRMT5 and 9 regulate monomethylation followed by symmetric dimethylation of arginine. PRMT7 is a type III PRMT that catalyzes only monomethylation. Among type I PRMTs, PRMT1 is known to perform 75% of type I activity in mammalian cells (Tang et al., 2000).

We have previously demonstrated that PRMT1 is essential for postnatal brain development, since *Prmt1*^flox/flox^*;Nes-Cre* neural stem cell-specific protein arginine methyltransferase 1 knockout (CKO) mice exhibit severe hypomyelination as well as astroglial and microglial activations (Hashimoto et al., 2016, 2021b). Furthermore, CKO mice had deregulation of neuronal glycan expression (Hashimoto et al., 2020). These studies showed that PRMT1 is important for various regulatory systems of neuronal and glial development in the CNS (Hashimoto et al., 2021a). A previous study on the role of PRMT1 in embryonic NSCs has shown that *Prmt1* knockdown in NSCs suppressed differentiation to an astrocyte lineage (Honda et al., 2017). Similarly, a recent report showed that *Prmt1* knockdown in primitive NSCs derived from mouse embryonic stem cells spontaneously expressed neuronal proteins such as NeuroD1, indicating that PRMT1 is essential for the maintenance of neural stemness (Chen et al., 2021). Although PRMT1 shows significantly higher expression in NSCs than differentiated neurons or astrocytes (Honda et al., 2017), a knockout study to identify the role of PRMT1 in embryonic NSCs proliferation and stemness is lacking. Moreover, it is necessary to evaluate PRMT1 deletion in NSCs in *in vivo* settings.

Here, to clarify the importance of PRMT1 in the proliferation and stemness of NSCs, we examined NSCs derived from Nestin-Cre mediated *Prmt1*-deficient mouse embryo (*Prmt1*^flox/flox^*;Nes-Cre*) both *in vivo* and *in vitro*. We found that NSCs in PRMT1-deficient embryonic brains proliferated normally *in vivo*. However, PRMT1-deficient NSCs showed reduced neurosphere formation and limited survival *in vitro*. NSCs cultured under hypoxic conditions modestly improved neurosphere formation but were PRMT1 independent. PRMT1 deficiency upregulated the accumulation of p21 and p53, which would be the main cause of the reduced proliferation and apoptosis in PRMT1-deficient NSCs.

## Materials and methods

### Animals

Nestin-Cre mediated *Prmt1*-deficient mice (*Prmt1*^flox/flox^*;Nes-Cre*) (C57BL/6 background) were generated as previously described (Hashimoto et al., 2016). *Prmt1*^flox/flox^*;Nes-Cre* mice are the CKO group and *Prmt1^flox/flox^* or *Prmt1^flox/wt^* mice are used as the control group throughout the study. On the following day of mating, the presence of a vaginal plug in the female mouse is defined as embryonic day 1 (E1). All animal experiments were carried out in accordance with and approved by the Institutional Animal Experiment Committee of Gifu University.

### RNA extraction and quantitative reverse transcription polymerase chain reaction

Total RNA was extracted from the forebrain of E14 mice and NSCs using ISOGEN II (Nippon Gene, Ltd., Tokyo, Japan #311-07361) and Ethachinmate (Nippon Gene, #312-01791). Total RNA was reverse-transcribed with ReverTra Ace qPCR RT Master Mix with gDNA Remover (Toyobo Co., Ltd., Osaka, Japan #FSQ-301). The relative gene expression level was determined by SYBR Green-based quantitative reverse transcription polymerase chain reaction (RT-PCR) (Bio-Rad Laboratories, Hercules, CA, USA #CFB3120EDU). The expression levels of the target genes were corrected for those of glyceraldehyde-3-phosphate dehydrogenase (*Gapdh*) and *β-Actin* expression levels using the *ddCt* method. The primer sequences are listed in Supplementary Table 1.

### *In vivo* 5-bromo-2′-deoxyuridine labeling and immunohistochemistry

**FIGURE 1:**
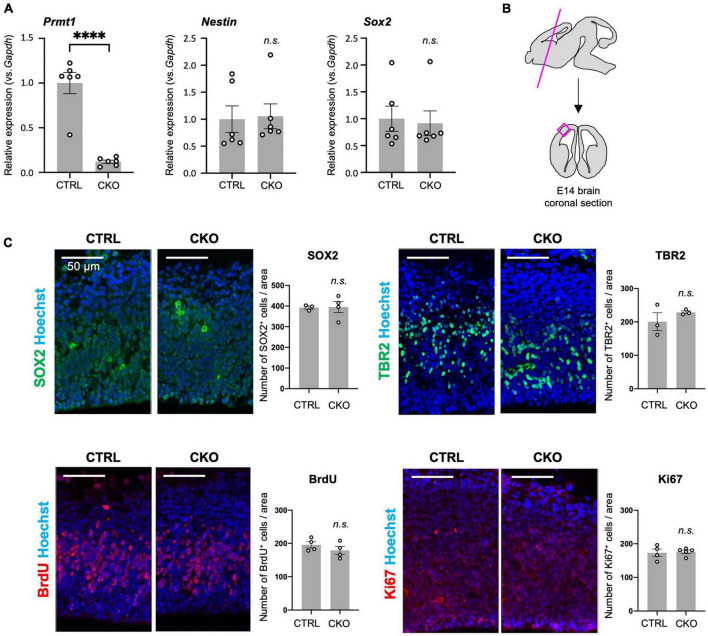
Protein arginine methyltransferase 1 (PRMT1)-deficient neural stem cells (NSCs) show substantial proliferation in the fetal cortices. **(A)** Relative expression of *Prmt1*, *Nestin*, and *Sox2* normalized to glyceraldehyde-3-phosphate dehydrogenase (*Gapdh*) in the forebrains of control (CTRL, *Prmt1^flox/flox^*) and neural stem cell-specific protein arginine methyltransferase 1 knockout (CKO) (*Prmt1^flox/flox^*;*Nes-Cre*) mice at embryonic day 14 (E14) analyzed by quantitative reverse transcription polymerase chain reaction (qRT-PCR). Data are shown as mean ± SEM (*n* = 6 animals) and were analyzed by a two-tailed Student’s *t*-test. ^****^p < 0.0001. **(B)** Schematic illustration of the E14 brain area for histological analyses. The magenta line is the plane for the sections and the areas for the observation and signal quantifications in panel **(C)** are indicated as a magenta square. The average cell count from both left and right cerebral cortices from a single section was used for cell count comparison in panel **(C)**. **(C)** Representative images of immunohistochemistry for SOX2, TBR2, 5-bromo-2′-deoxyuridine (BrdU), and Ki67 with Hoechst nuclear staining of control (CTRL) and CKO cerebral cortices at E14. The quantification data are shown as mean ± SEM (*n* = 3–5 animals). Scale bars, 50 μm.

Pregnant females at E14 were injected intraperitoneally with 5-bromo-2′-deoxyuridine (BrdU) (100 mg/kg body weight; Sigma-Aldrich, St. Louis, MO, USA #B5002). After 1 h, the females were sacrificed by cervical dislocation and the embryos were harvested. The embryonic heads were removed, fixed with 4% paraformaldehyde (PFA) overnight, and embedded in an O.C.T. compound (Tissue-Tek, Torrance, CA, USA #4583). Of note, 10-μm cryosections were performed heat-induced epitope retrieval with citrate buffer (pH 6.0). In case of BrdU staining, the sections were further incubated with 2N HCl. Sections were stained with primary antibodies after blocking with a 10% donkey serum donor herd (Millipore, Burlington, MA, USA #S30) in PBS or 10% normal goat serum (Sigma-Aldrich #G6767 and Jackson Immuno Research, West Grove, PA, USA #005-000-121) in PBS. The following antibodies and reagents were used: anti-Sox2 (Santa Cruz, Dallas, TX, USA #sc17320; 1:200), anti-BrdU (Novus Biologicals, Centennial, CO, USA NB500-169; 1:500), anti-TBR2 (Abcam, Cambridge, UK #ab23345; 1:100), anti-Ki67 (Thermo Fisher Scientific, Waltham, MA, USA #14-5698-82; 1:50), goat anti-rat IgG (H + L) cross-absorbed secondary antibody, Alexa Fluor 568 (Thermo Fisher Scientific #A-11077; 1:1,000), biotinylated rabbit anti-goat IgG antibody (H + L) (Vector, Newark, CA, USA #BA-5000; 1:200), biotinylated goat anti-rabbit IgG antibody (H + L) (Vector #BA-1000; 1:200), and Alexa Fluor 488-conjugated Streptavidin (Jackson Immuno Research, West Grove, PA, USA #016-540-084; 1:1,000). Cell nuclei were stained with Hoechst 33342 (Nacalai Tesque, Inc., Kyoto, Japan #04915-81 and FUJIFILM Wako Pure Chemical Corporation, Osaka, Japan #346-07951; 0.5 μg/ml). Fluorescence images were obtained with a fluorescence microscope (BIOREVO BZ-X710 and BZ-X810, Keyence, Osaka, Japan). Cell counting was performed using three to five mice per genotype and the same size areas were applied to manual cell counting using the ImageJ software. The area for cell counting is shown in Figure 1B. Each staining was performed in two sections from each animal and cell counting was performed using one of the two sections. The average count from both cortical sides was used for the graph and statistical analysis shown in Figure 1C.

### Isolation, culture, and treatments of neural stem cells

Neurosphere culture was performed referring to previously published protocol with some modifications (Hirabayashi et al., 2009). In detail, the forebrain regions of E14 mice were dissociated into single-cell suspensions with Neural Tissue Dissociation Kit (P) (Miltenyi Biotech, Gladbach, Germany #130-092-628). The cell suspension was passed through a 40-μm cell strainer (Corning, NY, USA #431750). Cells from individual embryos, 2 × 10^6^ cells/uncoated T25 flask, were cultured in 5 ml of proliferation media consisting of DMEM/Ham’s F-12 (FUJIFILM Wako Pure Chemical Corporation, #042-30555) with insulin, recombinant human (5 μg/ml; SAFC Biosciences, Lenexa, KS, USA #91077C), transferrin (Apo), from human blood (100 μg/ml; FUJIFILM Wako Pure Chemical Corporation, #205-18121), progesterone (6.4 ng/ml; Sigma-Aldrich, #P8783), Putrescine dihydrochloride (16 μg/ml; Sigma-Aldrich, #P5780) and sodium selenite (26 ng/ml; Sigma-Aldrich, #S5261), B27 supplement (Thermo Fisher Scientific, Waltham, MA, USA #17504-044), penicillin-streptomycin mixed solution (Nacalai Tesque, #26253-84), bFGF (25 ng/ml; PeproTech, Cranbury, NJ, USA #450-33), and EGF (25 ng/ml; Thermo Fisher Scientific, #PMG8041). Heparin is not added to the neurosphere culture. For cell proliferation, bFGF and EGF were added every 3 or 4 days. Cells were incubated at 37°C in 5% CO_2_ and 21% O_2_ for normoxic culture. For hypoxic culture, cells were maintained in 2.5% O_2_ using Anaero Pack (Mitsubishi Gas Chemical, Tokyo, Japan #A-07). Primary neurosphere diameter was measured on day 5 *in vitro* using the ImageJ software. More than 100 spheres in three to six images obtained from one to two flasks were measured in each condition.

### *In vitro* 5-bromo-2′-deoxyuridine labeling and immunocytochemistry

The primary spheres were dissociated and cells were seeded on poly-D-lysine-coated coverslips in 24-well plates. After 2 days, cells were labeled BrdU (10 μM; Sigma-Aldrich, #B5002) for 6 h, fixed with 4% PFA, treated with 2N HCl, and blocked with 10% goat serum donor herd (Sigma-Aldrich, #G6767) in 0.1% TritonX-100/PBS for 1 h. Primary antibodies were applied overnight at 4°C followed by secondary antibodies for 1 h at room temperature. The following antibodies were used: anti-BrdU (Novus Biologicals, NB500-169; 1:200), anti-cleaved caspase-3 (Asp175) (Cell Signaling Technology, Danvers, MA, USA #9661; 1:400), goat anti-rat IgG (H + L) cross-absorbed secondary antibody, Alexa Fluor 568 (Thermo Fisher Scientific, #A-11077: 1:1,000), Alexa Fluor 488-affinipure donkey anti-rabbit IgG (H + L) (Jackson Immuno Research, #016-540-084; 1:1,000). Cell nuclei were stained with Hoechst 33342. Fluorescence images were obtained with a fluorescence microscope (BIOREVO BZ-X710 and BZ-X810, Keyence). BrdU^+^ cell and cleaved caspase-3^+^ cells were counted using eight independent fields of 40× objectives per each group and were applied to manual cell counting using the ImageJ software.

### Western blot analysis

The NSCs were homogenized by sonication in ice-cold TNE buffer (20 mM Tris–HCl pH 7.5, 150 mM NaCl, 1 mM EDTA) with 0.2% Nonidet P-40 and protease inhibitor mixture (Nacalai Tesque, #25955-11). Protein concentrations were determined by Bradford assays with XL-Bradford (APRO Life Science Institute, Tokushima, Japan #KY-1030). Proteins were separated by 8% SDS-PAGE and blotted onto polyvinylidene fluoride (PVDF) membranes (Millipore, #IPVH00010). The membranes were blocked with 3% skim milk in TBS containing 0.1% of Tween-20 (TBS-T), and incubated with primary antibody and then with HRP-conjugated secondary antibodies which were diluted with 1 and 0.5% skim milk in TBS-T. The following primary antibodies were used: anti-asymmetric dimethyl arginine, Asym26 (Epicypher, Durham, NC, USA #13-0011; 1:500), anti-p53 (1C12) (Cell Signaling Technology, #2524; 1:1,000), anti-phospho-p53 (FP3.2) (Santa Cruz Biotechnology, #sc-51690; 1:200), anti-p21 (EPR18021) (Abcam, Cambridge, UK #ab188224; 1:1,000), anti-H2AX (Sigma-Aldrich, #07-627; 1:1,000), anti-phospho-H2AX (Ser139) (Sigma-Aldrich, #05-636; 1:500), anti-PRMT1 (Millipore, #07-404; 1:1,000), and anti-Hsp70 (Bio-Legend, San Diego, CA, USA #648001; 1:500). Visualization was performed by chemiluminescent detection using ImmunoStar Zeta (FUJIFILM Wako Pure Chemical Corporation, #295-72404) or Immobilon ECL Ultra Western HRP Substrate (Millipore, WBULS0100). Immunoreactive images were captured by LAS-3000 (Fujifilm, Tokyo, Japan).

### Statistical analysis

**FIGURE 2:**
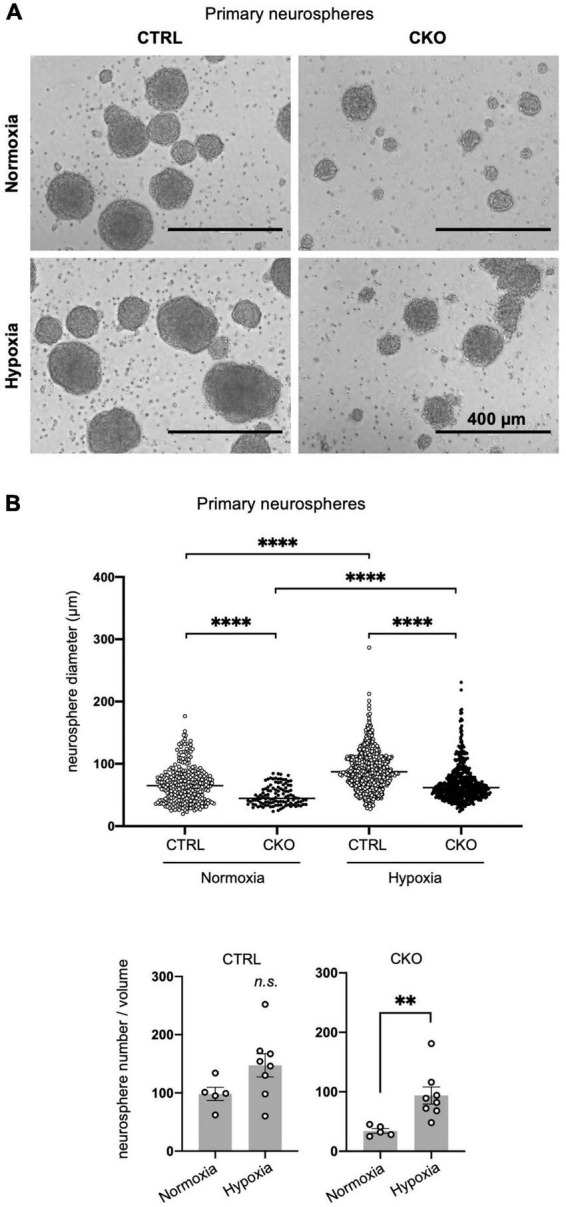
Decreased sphere formation of protein arginine methyltransferase 1 (PRMT1)-deficient neural stem cells (NSCs). **(A)** Representative phase contrast images of primary neurospheres. NSCs were obtained from E14 forebrains from control (CTRL, *Prmt1*^flox/flox^** and *Prmt1^flox/wt^*) and neural stem cell-specific protein arginine methyltransferase 1 knockout (CKO) (*Prmt1*^flox/flox^*;Nes-Cre*) mice and were cultured for 5 days *in vitro* to obtain neurospheres. Cell culture was performed under normoxia (21% O_2_) in upper panels and hypoxia (2.5% O_2_) in lower panels. Scale bars, 400 μm. **(B)** Neurosphere diameter (upper panel) and neurosphere number (lower panel) were measured on day 5 *in vitro* under normoxia and hypoxia. Neurosphere diameter data (*n* > 100) are shown as mean ± SEM and were analyzed by the Kruskal–Wallis test followed by Dunn’s multiple comparisons test. ^****^p < 0.0001. Sphere number data (*n* > 5) are shown as mean ± SEM and were analyzed by a two-tailed Student’s *t*-test. ^**^p < 0.01.

Results are shown as mean ± SEM. Normality assessment was not applied to all data. Data were analyzed using a two-tailed Student’s *t*-test for comparisons of two groups. Neurosphere diameter data (Figure 2B, upper panel) was analyzed using the Kruskal–Wallis test followed by Dunn’s multiple comparison test. Significance was considered at *p* < 0.05. Statistical analysis was performed using Prism 8 for macOS [Version 8.4.3 (471)].

## Results

### Normal neural stem cell proliferation in *Prmt1*^flox/flox^*;Nes-Cre* embryonic brains

Although PRMT1 has been shown to be essential for neurons and glial cells (Hashimoto et al., 2016, 2021a), it was unclear whether these origins, Nestin^+^ NSCs, normally survive and proliferate after PRMT1 deficiency in the embryonic brain. Therefore, we measured the mRNA expression of NSC-specific genes by qRT-PCR. *Prmt1* was shown to be markedly downregulated in CKO forebrains compared to control tissues (Figure 1A). Both *Nestin* and *Sox2*, well-characterized NSC markers, were expressed at normal levels in CKO tissues, suggesting that a proper NSC population is maintained in the CKO forebrains (Figure 1A). We further verified by immunostaining that SOX2-positive cells were distributed in the cerebral cortices in both control and CKO (Figure 1C). To investigate the cell proliferation rate in the embryonic cerebral cortices, we performed BrdU assays at E14. At 1 h after BrdU injection into the pregnant dams, the number of proliferating cells incorporated with BrdU was the same between control and CKO in the cerebral cortices at E14 (Figure 1C). In consistent with this, the number of Ki67-positive proliferating cells was not affected in CKO (Figure 1C). We also found that the number of TBR2-positive intermediate progenitor cells, which is another proliferating cell type besides NSC, was comparable between CKO and control (Figure 1C). Taken together, these data suggest that the PRMT1-deficient NSC population, proliferation rate, and distribution are normal in embryonic cerebral cortices at E14.

### Decreased proliferation and survival of protein arginine methyltransferase 1-deficient neural stem cells cultured as neurospheres under different oxygen concentrations

In our Nestin-Cre-driven PRMT1 knockout mice, PRMT1 is depleted not only in NSCs but also in all types of cells differentiated from NSCs. Thus, it is difficult to discriminate the role of PRMT1 in NSCs from some secondary effects on NSCs from surrounding cells in *in vivo* settings. To simply characterize the role of PRMT1 in NSC proliferation, we cultured NSCs derived from mouse forebrains and evaluated their proliferation by neurosphere-forming assays. To our surprise, PRMT1-deficient NSCs did not form many primary neurospheres on day 5 *in vitro* (Figure 2A). Furthermore, neurosphere diameter, which is an indicator of NSC proliferation, was significantly decreased in the CKO normoxia culture condition (Figure 2B, upper panel). These results are different from our observation that NSCs showed normal proliferation *in vivo* (Figure 1C).

In the embryonic brain, NSCs are considered to be in an environment with relatively low oxygen availability (Mohyeldin et al., 2010). Additionally, oxygen tension in the embryonic brain has been shown to be low by using a hypoxyprobe, pimonidazole (Mutoh et al., 2012), suggesting that lower oxygen concentrations would be more physiological. Therefore, we next tried the same neurosphere assay under hypoxic conditions (2.5% O_2_). Compared to atmospheric oxygen concentration (21% O_2_), NSCs appeared to survive and increased better in hypoxic culture in both control and CKO (Figures 2A,B, lower panel), although the diameter of the neurosphere was still significantly lower in PRMT1-CKO than in control groups even in hypoxia (Figure 2B, upper panel). It has been suggested that Wnt/β-catenin signaling positively regulates NSC proliferation under hypoxia (Braunschweig et al., 2015). Since both control and CKO NSCs responded to decreased oxygen concentration and increased cell proliferation, it is suggested that growth control signaling was upregulated in a PRMT1-independent manner. Therefore, the following experiments were performed only under normoxic conditions. Since we could not obtain enough secondary neurospheres suitable for the following biochemical assays in case of CKO, we decided to perform the following assays using primary neurospheres.

### Reduced proliferation and viability of protein arginine methyltransferase 1-deficient neural stem cells

**FIGURE 3:**
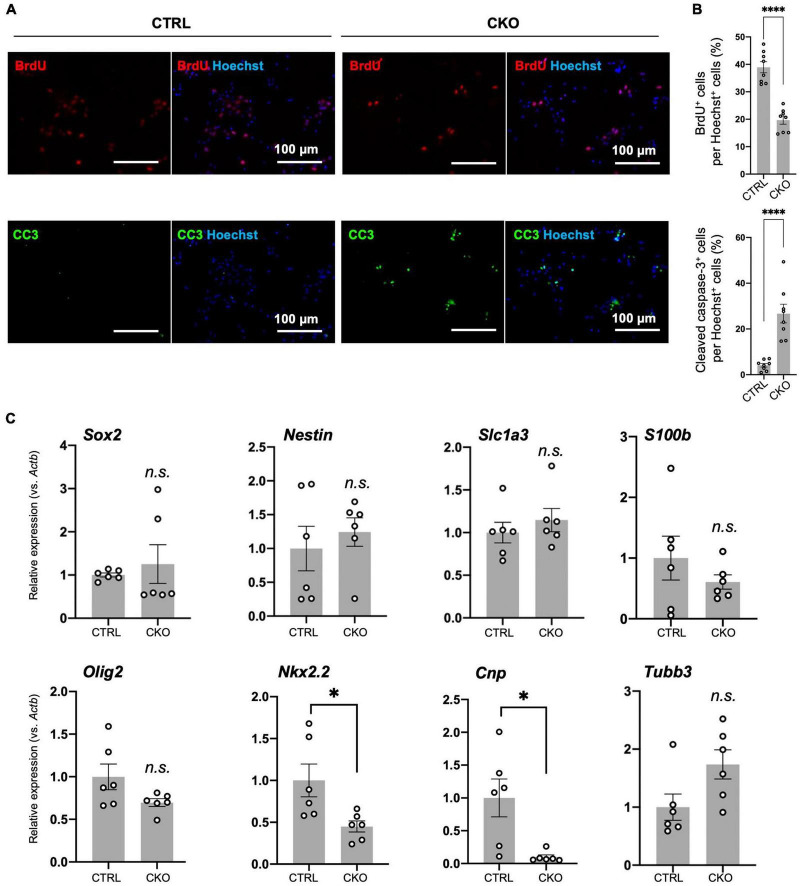
Protein arginine methyltransferase 1 (PRMT1)-deficient neural stem cells (NSCs) have reduced proliferation and viability in culture, but their differentiation potential has not changed. **(A)** Representative images of immunocytochemistry for 5-bromo-2′-deoxyuridine (BrdU) (top), cleaved caspase-3 (CC3) (bottom). Each image is merged with Hoechst nuclear staining (right side of each panel). Day 2 NSCs obtained after dissociation of the primary neurosphere were treated with BrdU for 6 h of incorporation and were used for the staining. Scale bars, 100 μm. **(B)** The quantification data of BrdU- or cleaved caspase-3-positive cells are shown as mean ± SEM (*n* = 8 fields from two independent cultures) and were analyzed by a two-tailed Student’s *t*-test. ^****^p < 0.0001. **(C)** Relative expression of *Sox2*, *Nestin*, *Slc1a3* (encoding GLAST), *S100b*, *Olig2*, *Nkx2.2*, *Cnp*, and *Tubb* normalized to *Actb* in primary neurosphere at culture day 6 analyzed by quantitative reverse transcription polymerase chain reaction (qRT-PCR). Data are shown as mean ± SEM (*n* = 6) and were analyzed by a two-tailed Student’s *t*-test. *p < 0.05.

To investigate the mechanism by which PRMT1 deficiency deregulates cell proliferation, we first performed BrdU incorporation assays with dissociated primary neurospheres. At 6 h after BrdU treatment, compared to control cells, CKO NSCs exhibited a decrease in BrdU-positive cells under normoxia, suggesting that cell cycle progression was affected by PRMT1 deficiency in NSCs (Figures 3A,B). Furthermore, we have found that the number of cleaved caspase-3 positive cells was significantly increased in CKO NSCs (Figures 3A,B). From these data, it was shown that the loss of PRMT1 in NSCs provoked both cell cycle deregulation and apoptosis.

Decreased proliferation of NSCs commonly suggests its increased tendency to spontaneous differentiation (Braunschweig et al., 2015). To test this, we measured the expression of stemness marker mRNA (*Sox2, Nestin*) and differentiation markers (*Olig2, Nkx2.2, Cnp, Slc1a3* (encoding GLAST/EAAT1), *S100b*, and *Tubb3*) by qRT-PCR. *Olig2* and *Nkx2.2* are essential genes for oligodendrogenesis (Qi et al., 2001; Takebayashi et al., 2002). *Cnp* encodes 2′,3′-cyclic nucleotide-3′-phosphodiesterase and is known to be abundantly expressed in oligodendrocyte lineage cells (Yu et al., 1994). *Slc1a3* is an astrocyte-specific glutamate transporter and shows early expression in immature astrocytes (Molofsky et al., 2012). Since *Slc1a3* is also suggested to be expressed in oligodendrocyte precursors (Molofsky et al., 2012), we also tested another astrocytic gene called *S100b* (Molofsky et al., 2012). *Tubb3* encodes βIII-tubulin and is highly expressed in differentiated neurons. As a result, most of these genes were expressed at the same level between the control and CKO (Figure 3C). It is noteworthy that *Nkx2.2* and *Cnp* were downregulated in CKO (Figure 3C), which may explain the decreased potential for oligodendrocyte differentiation resulting in hypomyelination due to PRMT1 deficiency (Hashimoto et al., 2016). These results suggest that PRMT1 deficiency does not largely influence the stemness of NSCs, but rather severely affects the proliferation and survival of NSCs.

### Cell cycle arrest, p53 accumulation, and apoptosis in protein arginine methyltransferase 1-deficient neural stem cells

**FIGURE 4:**
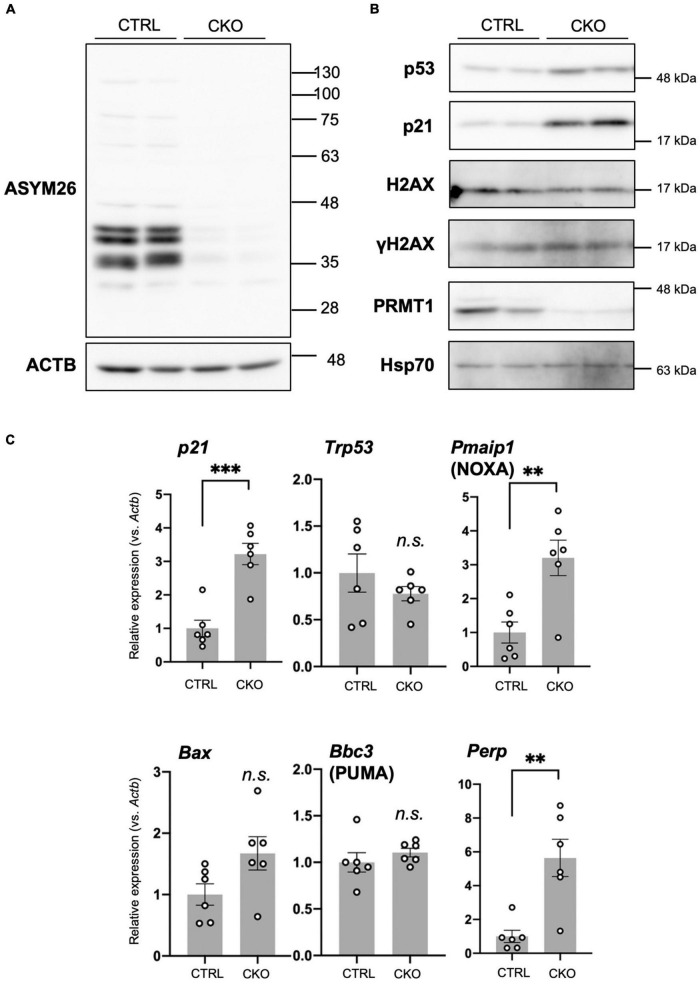
Cell cycle arrest, p53 accumulation, and apoptosis in protein arginine methyltransferase 1 (PRMT1)-deficient neural stem cells (NSCs). **(A)** Asymmetric dimethyl arginine levels in primary neurosphere at culture day 6 analyzed by Western blots using Asym26. **(B)** Expression of indicated proteins in primary neurosphere at culture day 6 analyzed by Western blots. **(C)** Relative expression of indicated genes normalized to *Actb* in primary neurosphere at culture day 6 analyzed by quantitative reverse transcription polymerase chain reaction (qRT-PCR). Data are shown as mean ± SEM (*n* = 6) and were analyzed by a two-tailed Student’s *t*-test. ^**^p < 0.01, ^***^p < 0.001.

Loss of PRMT1 in NSCs induced dramatic suppression of asymmetric dimethyl arginine of proteins (Figure 4A), suggesting that PRMT1 is the major enzyme for this modification in embryonic NSCs. Next, we focused on how this change affected elevated apoptosis and decreased proliferation in PRMT1-deficient NSCs. p53, a well-known tumor suppressor transcription factor, responds to various stresses and regulates both cell cycle repression and apoptosis in cancer and non-cancer cell types (Polager and Ginsberg, 2009). p53 is constantly degraded by mouse double minute 2 homolog (MDM2)-mediated proteasome pathways, while it accumulates under stresses (Polager and Ginsberg, 2009). To assess the accumulation of p53, we checked the level of p53 protein using protein extracts from primary neurospheres of CKO mice. We found that p53 levels were upregulated in the CKO neurospheres compared to the control (Figure 4B). On the other hand, the level of *Trp53* mRNA, which encodes p53, was unchanged in CKO NSCs (Figure 4C). These results suggest that the p53 protein was accumulated in PRMT1-deficient NSCs.

Furthermore, by Western blotting, we showed that p21, a cyclin-dependent kinase (CDK) inhibitor, was significantly increased in CKO NSCs (Figure 4B). Concomitantly, the level of *p21* mRNA increased in CKO NSCs (Figure 4C). p21 is one of the major p53 target genes and controls cell cycle arrest (Polager and Ginsberg, 2009). Taken together, our data suggest that p53 accumulation induced p21 transcription and led to the growth arrest of PRMT1-deficient NSCs.

In CKO NSCs, apoptosis was significantly induced (Figures 3A,B). The Bcl-2 family proteins play a crucial role in the induction or repression of apoptosis (Gross and Katz, 2017). Among pro-apoptotic members of the Bcl-2 family, we found that *Pmaip1*, encoding NOXA, was markedly upregulated in CKO NSCs (Figure 4C). Other pro-apoptotic Bcl-2 families such as *Bbc3* (encoding PUMA) and *Bax* did not show such a difference. However, in case of *Bax*, it is of note that CKO NSCs showed a higher trend than control NSCs (Figure 4C). Furthermore, *Perp*, one of the target genes for p53 and known as an apoptosis mediator (Attardi et al., 2000; Marques et al., 2005), increased significantly in CKO NSCs compared to the control group (Figure 4C).

Since DNA damage is one of the main stressors that induce p53 accumulation, we also tested whether DNA damage is a trigger for the upregulation of p53 in CKO NSCs. The phosphorylated histone H2AX (γH2AX) is a useful marker of DNA damage (Mah et al., 2010). Western blot analyses showed that γH2AX levels did not increase in CKO NSCs (Figure 4B), suggesting that DNA damage would not be the reason for the accumulation of p53 in PRMT1-deficient NSCs.

Taken together, after PRMT1 depletion in NSC, p21 showed a striking accumulation that caused cell cycle arrest. In parallel, p53 was accumulated followed by the induction of apoptosis mediators, including *Pmaip1* and *Perp*. Although p21 is generally known to suppress apoptosis and contribute to cellular repair, our data imply that the p53-mediated pro-apoptotic signals exceeded this effect in the case of PRMT1-deficient NSCs.

### Potential extracellular factors that support neural stem cell proliferation and survival in protein arginine methyltransferase 1-deficient brains

**FIGURE 5:**
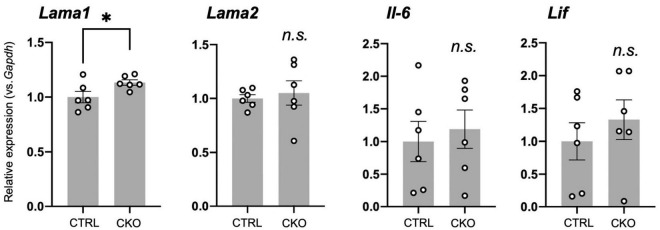
Potential extracellular factors supporting neural stem cell (NSC) proliferation and survival in protein arginine methyltransferase 1 (PRMT1)-deficient brains. Relative expression of *Lama1*, *Lama2*, *Il-6*, and *Lif* normalized to glyceraldehyde-3-phosphate dehydrogenase (*Gapdh*) in the forebrains of control and neural stem cell-specific protein arginine methyltransferase 1 knockout (CKO) E14 analyzed by quantitative reverse transcription polymerase chain reaction (qRT-PCR). Data are shown as mean ± SEM (*n* = 6 animals) and were analyzed by a two-tailed Student’s *t*-test. *p < 0.05.

We have previously demonstrated increased astrocytes and microglia as well as upregulated inflammatory responses in neonatal brain cortices of CKO mice (Hashimoto et al., 2021b). Therefore, we ask whether the cellular and molecular changes in the brain provided an ideal microenvironment for the survival of NSCs in PRMT1 deficiency. Indeed, many extracellular factors are known to contribute to the survival, attachment, and proliferation of NSCs (Kazanis and ffrench-Constant, 2011). Thus, we performed qRT-PCR of PRMT1-deficient brains at E14, when the normal distribution of NSC was confirmed (Figure 1C). A major extracellular matrix protein, laminin, has been reported to provide signaling cues for NSC adhesion and proliferation (Li et al., 2014; Kim et al., 2021). Laminin is a heterotrimeric protein, and astrocytes express laminins-111 (α1β1γ1) and -211 (α2β1γ1) (Yao et al., 2014). While α2 subunit (*Lama2*) was normal, the α1 subunit (*Lama1*) was significantly highly expressed in the brain cortices of CKO (Figure 5), indicating that laminin is one of the potential factors for the survival and proliferation of CKO NSCs. Among the main CNS cytokines, IL-6 secreted by astrocytes is known to stimulate NSC proliferation (Wang et al., 2011). LIF is also known to stimulate the formation of neurospheres of adult NSCs (Bauer, 2009). However, *Il-6* and *Lif* did not show a significant difference in the embryonic PRMT1-CKO brains (Figure 5). Taken together, our data indicate a potential contribution of the extracellular matrix to the survival/proliferation of PRMT1-deficient NSCs in the brain microenvironment.

## Discussion

### Effects of protein arginine methyltransferase 1 loss in neural stem cells *in vivo* and *in vitro*

In this study, we have shown that cultured PRMT1-deficient NSCs derived from E14 mouse forebrains showed deregulated cell proliferation, apoptosis, and accumulation of p21 and p53. However, on the other hand, we found that PRMT1-deficient NSCs *in vivo* showed normal proliferation evaluated by BrdU incorporation, Ki67-positive cell number, and distribution of NSCs in the cerebral cortices at the same stage of embryonic development as preparation of NSC culture. To address this discrepancy, we tried culturing NSCs under hypoxic conditions, which are considered to be close to the physiological oxygen environment of embryonic brains. The primary neurospheres of the CKO brain increased the number and diameter of the sphere by lowering the oxygen concentration; however, hypoxia did not give enough recovery to the control level. Therefore, there may be some other *in vivo* factors necessary for the proliferation and survival of PRMT1-deficient NSCs.

In search for the reason for the discrepancy in PRMT-deficient NSC proliferation *in vivo* and *in vitro*, we focused on the possible upregulation of inflammatory signaling in the PRMT1-deficient brain. We have previously found that the aberrant increase and activation of astrocytes and microglia in the cerebral cortices of PRMT1-CKO after birth (Hashimoto et al., 2021b). In parallel, we have also shown the upregulation of various cytokine/chemokine-related genes and extracellular matrices in the neonatal CKO cortices (Hashimoto et al., 2021b). Previous research has demonstrated that astrocyte-conditioned media or IL-6 treatment induced NSC proliferation *in vitro* (Wang et al., 2011). Therefore, we sought to determine whether these cytokines from neighboring cells of NSCs, which are absent in culture systems, could be important for the proliferation of CKO NSCs. This prompted us to test the level of neurotrophic cytokines in the embryonic stages. However, we did not see higher trends of *Il-6* and *Lif* in the fetal brain of CKO (Figure 5). On the other hand, we found that *Lama1*, encoding α1 subunit of laminin, was significantly increased in the brain of CKO. Future rescue studies using adherent NSC culture over a laminin coat or the addition of laminin in a culture medium for CKO NSCs will be required to identify the precise mechanism. In addition, preliminary secretome analyses of embryonic brains are needed to fully cover the differences in extracellular signals that support the survival of CKO NSCs.

In addition to extracellular molecules, we should also consider the role of extracellular forces that are different between *in vitro* and *in vivo*. In the embryonic brain cortex, NSCs have a bipolar shape and are packed in the neurepithelium which has an apical–basal axis (Miyata, 2008). The mechanical tension in the environment to NSCs that are only emerged *in vivo* is suggested to be important for stem cell proliferation (Pathak et al., 2014; Kumar et al., 2017; Vining and Mooney, 2017). Thus, it is probable that PRMT1 deficiency using *Nestin-Cre* affects the extracellular forces, in addition to secretory molecules, from neighboring cells in the neurepithelium, leading to compensation of the NSC proliferation. While we have observed normal proliferation of NSCs in CKO cortices at E14, it is still possible that the proliferation potential changes during embryonic development due to the modification of both intracellular and extracellular signals by loss of PRMT1 *in vivo*.

### Molecular control of survival and proliferation of neural stem cells by protein arginine methyltransferase 1

In our study, upregulation of the p53-mediated apoptotic pathway at least partially explains the increased cell death of PRMT1-deficient NSCs. A previous trial of inducible PRMT1 loss in mouse embryonic fibroblasts (MEFs) demonstrated spontaneous DNA damage and decreased proliferation without inducing apoptosis (Yu et al., 2009). Although our data did not show DNA damage in PRMT1-deficient NSCs by evaluating γH2AX (Figure 4B), it would need more detailed chromosomal analyses. Therefore, it remains uncovered how PRMT1 loss leads to the accumulation of p53 in embryonic NSCs.

In terms of other members of the PRMT family and p53 signaling, a previous work demonstrated that PRMT1-p53 regulates epicardial invasion for normal heart development through PRMT1-mediated *Mdm4* splicing (Jackson-Weaver et al., 2020). Another study showed that PRMT5 is essential for NSC survival and proliferation through the generation of the functional *Mdm4* splicing variant which avoids aberrant accumulation of p53 (Bezzi et al., 2013). Our study found that p53 was accumulated at the protein level but not the mRNA level in CKO NSCs (Figures 4B,C), indicating that a similar p53 stabilization mechanism was activated by PRMT1 deficiency in NSCs. Therefore, our study highlights the importance of PRMT1 arginine methylation in NSCs in addition to PRMT5.

One limitation of our study is that we obtained the above data on p53 accumulation in primary neurospheres because we could not obtain enough secondary sphere population. Generally, neurosphere studies basically use secondary or tertiary neurospheres because they are groups of NSCs that have self-renewal capacity and are therefore suitable to NSC analyses. At least, we have confirmed that the primary neurospheres expressed relatively high levels of *Sox2* and *Nestin* both in CTRL and CKO (Figure 3C), suggesting that it would be meaningful to compare their proliferation capacity and molecular control.

In this study, we found arginine methylation of multiple proteins in primary neurospheres, and these were markedly suppressed by PRMT knockout (Figure 4A). These data suggest that PRMT1 is the major type I PRMT in NSCs. PRMT1 is also known as a popular epigenetic regulator because it methylates histone H4 arginine 3 (H4R3) (Wang et al., 2001). Meanwhile, asymmetrically methylated H4R3 (H4R3me2as) has not been detected in NSCs in the embryonic cortices (Chittka, 2010), implying that PRMT1 has non-histone targets in NSCs. While we did not focus here, NSC proliferation is essentially regulated by various growth signals, such as Wnt, MAPK, or Notch, as described elsewhere (Chenn and Walsh, 2002; Campos et al., 2004; Ables et al., 2011). Our previous data of RNA-seq of P0 cortices derived from CKO mice did not reveal any significant changes in these signals (Hashimoto et al., 2021b); however, it is possible that these signals are deregulated in cultured CKO NSCs because their proliferation is severely affected.

### Roles of protein arginine methyltransferase 1 in the proliferation and differentiation of neural stem cells

A previous work has demonstrated that the knockdown of PRMT1 in NSCs reduced the potential for differentiation specifically in an astrocyte lineage through *Gfap* transcriptional repression, suggesting that PRMT1 is essential for the regulation of astrocytic differentiation (Honda et al., 2017). Although we have also tried differentiation assays using CKO NSCs, unfortunately, they did not survive well during differentiation (data not shown), so we could not collect enough data on differentiated cells. In our experiment, we found that PRMT1-deficient NSCs expressed the normal level of *Slc1a3* (GLAST) and *S100b*, which indicates an intact astrocytic differentiation potential after PRMT1 loss. Therefore, it is implied that the deregulation of astrocytic differentiation by loss of PRMT1 occurs in a setting in which NSC differentiation was strongly induced.

Our previous study has shown that OLIG2-positive oligodendrocyte precursor cells (OPCs) or immature oligodendrocytes were specifically reduced in PRMT1-deficient neonatal brains (Hashimoto et al., 2016). In the present study, CKO NSCs expressed a lower level of *Nkx2.2* and *Cnp* compared to control NSCs, in accordance with our previous findings. Therefore, it is implied that the oligodendrocyte differentiation potential was already affected in PRMT1-deficient NSCs. In addition to this, as we have previously suggested, brain inflammatory changes in CKO mice could have an additional negative impact on the survival and differentiation of OPCs (Hashimoto et al., 2021b).

Several recent studies are aimed at modulating NSCs for transplantation at neuronal injury sites (Yousefifard et al., 2016; Marsh and Blurton-Jones, 2017; Kitagawa et al., 2022). Therefore, it is of great importance to explore a novel molecular control of NSC proliferation, survival, and differentiation. Our findings that PRMT1 is essential for NSC survival and proliferation emphasize that arginine methylation is vital for NSCs. Furthermore, the unexpectedly normal proliferation of PRMT1-deficient NSCs *in vivo* prompts us to identify some extracellular factors that could support the survival of NSCs. Further molecular studies will pave the way for new therapeutic strategies for neuronal injury.

## Data availability statement

The original contributions presented in this study are included in the article/Supplementary material, further inquiries can be directed to the corresponding author.

## Ethics statement

This animal study was reviewed and approved by the Gifu University.

## Author contributions

KT, AK, AY, and KI performed the experiments. MH and KT wrote the original manuscript. KM, CS-N, YK, AF, and TN revised the manuscript. All authors contributed to the article and approved the submitted version.

## Abbreviations

BrdU: 5-bromo-2 ′ -deoxyuridine
CKO: neural stem cell-specific protein arginine methyltransferase 1 knockout
CNS: central nervous system
E: embryonic day
NSC: neural stem cell
OPCs: oligodendrocyte precursor cells
PRMT1: protein arginine methyltransferase 1
qRT-PCR: quantitative reverse transcription polymerase chain reaction.
